# Atypical giant proliferating cystic pilomatrixoma: A case review

**DOI:** 10.1177/2050313X251320195

**Published:** 2025-02-27

**Authors:** Kerman Sekhon, Yvette Miller-Monthrope, Christian Murray, Michael Weinberg

**Affiliations:** 1 Temerty Faculty of Medicine, University of Toronto, Toronto, ON, Canada; 2 Dermatology Division, Department of Medicine, Women’s College Hospital, Toronto, ON, Canada; 3 Plastic Surgery Division, Department of Surgery, Trillium Health Centre, Mississauga, ON, Canada

**Keywords:** Pilomatrixoma, pilomatricoma, calcifying epithelioma of Malherbe, atypical pilomatrixoma, giant pilomatrixoma, cystic pilomatrixoma, proliferating pilomatrixoma

## Abstract

Pilomatrixoma, or calcifying epithelioma of Malherbe, is a rare and benign tumour. Typical presentation of pilomatrixoma is solitary, firm lesions found in the head and neck regions. Pilomatrixoma are often misdiagnosed due to their clinical presentation, which is synonymous with other cutaneous pathology. The reported and true incidence of pilomatrixoma are 1% and 16%, respectively. Cystic pilomatrixoma is a unique variant that presents without the pathognomonic feature of a firm lesion. This study presents the case of a 52-year-old man with a post-auricular pilomatrixoma. It is erythematous, soft, mobile, and 4 cm in diameter. Its size categorizes the lesion as a giant pilomatrixoma. These pilomatrixoma pose a diagnostic challenge as they mimic malignant lesions. It was highly erythematous and mimicked Merkel cell carcinoma in on initial presentation. It was a sebum-filled lesion, which is quite atypical for pilomatrixoma. A previous study finds that only three cases of cystic pilomatrixoma have been reported in the literature previously. This case study presents the case of an atypical variant of a rare tumour. There is a paucity in the literature on cystic pilomatrixoma. Giant proliferating cystic pilomatrixoma has not previously been described in a case report in the literature, as to our knowledge. This study highlights the diversity in the clinical presentations of pilomatrixoma to improve our clinical sensitivity to pilomatrixoma.

## Introduction

Pilomatrixoma, or calcifying epithelioma of Malherbe, is a rare and benign tumour.^1^ The reported incidence of pilomatrixoma is 1%, among benign lesions.^2^ However, it is predicted that the true incidence is close to 16%.^3^ Pilomatrixoma often goes undiagnosed, a significant reason for this is its clinical presentation, which is synonymous with other dermal and subcutaneous masses such as epidermal cysts and trichilemmal cysts.^3–5^ Pilomatrixoma will typically present as a solitary, firm, non-tender, and mobile lesion in the head and neck region.^5^ In addition to its several other pathognomonic features, such as tent sign and teeter-totter sign, is the characteristic finding of a firm lesion.^4,6,7^ This clinical characteristic is absent in the cystic pilomatrixoma variant presented in our case report, which further confounds its clinical diagnosis.

### Clinical presentation and risk factors

Pilomatrixoma is a benign tumour originating in the hair follicle and typically presents in the head, neck, scalp, and upper trunk.^1,8,9^ Post-auricular pilomatrixoma has been reported previously.^10,11^ It is usually found as a single lesion; however, some cases of pilomatrixoma clusters have been reported.^12–14^ Multiple pilomatrixomas are associated with certain genetic disorders, such as myotonic dystrophy, familial adenomatous polyposis (Gardner Syndrome), or Rubinstein-Taybi syndrome.^15–17^ Typically it is less than 1 cm, or up to 3 cm in diameter.^9^ Pilomatrixoma measuring 4 cm or greater are considered ‘giant’.^18,19^ It may be regular or irregular in shape.^20^

A key characteristic is its firm consistency, due to the fact that pilomatrixoma are calcifying lesions.^21^ In addition, calcification results in an angulated lesion, or the ‘tent sign’.^6^ Another sign seen with pilomatrixoma is the ‘teeter-totter’, reproduced by palpating one edge of the lesion with subsequent protrudence of the opposite edge.^21^ Recurrence of the lesion post-excision is rare.^22^ Recurrence rates have been reported by various sources between 0% and 5%.^1,14,23^

Previous case reports on pilomatrixoma centre around Caucasians. A recent review finds a slightly higher proportion of women with pilomatrixoma than men.^1^ A bimodal distribution has been noted in terms of prevalence by age, with pilomatrixoma being more common in younger, 5- to 15-year-old, and then older, 50- to 65-year-old, individuals.^24^ Mean age of onset is 16 years old.^24^

### Pathogenesis

A mutation within the hair follicular matrix cells is the source of pilomatrixoma.^25^ It is postulated that the development of pilomatrixoma involves the Wnt signalling pathway.^25^ The oncogene BCL-2, which represses apoptosis processes, has been implicated in pilomatrixoma.^26^ Furthermore, CTNNB1, causing LEF-1 or B-catenin dysregulation, has also been implicated.^27^

### Rationale

Pilomatrixoma poses a significant diagnostic challenge. The low incident rate, in combination with a clinical presentation that is homogenous with other dermatologic pathology, presents considerable concern for misdiagnosis. This case report further elucidates the need to recognize and diagnose pilomatrixoma, as we present a unique case of pilomatrixoma. This study illustrates an atypical presentation of pilomatrixoma; proliferating giant cystic pilomatrixoma is an uncommon presentation of pilomatrixoma and there is a paucity of literature on this variation. This case report will contribute to existing knowledge on proliferating giant cystic pilomatrixoma.

## Case presentation

A 52-year-old man presented to a tertiary care clinic in Ontario, Canada, with a 3.3 cm erythematous post-auricular mass, which had grown gradually over a period of 6 months. He had no relevant past medical history. On examination, the patient had a large nodule post-auricularly, that was soft in consistency and had a bluish hue on an erythematous base of skin. There was an ulcerating core and glistening quality to its superior-most edges. The lesion was originally thought to be a Merkel cell carcinoma due to its highly erythematous clinical presentation. During biopsy, sebum fluid discharged from the lesion.

Histopathology revealed a proliferating pilomatrixoma. There was a recurrence of the lesion. The recurring lesion was 4 cm in diameter, erythematous, non-tender, mobile, and with well-defined borders. It was also soft in consistency. The patient was treated with antibiotics post-operatively, as swelling persisted chronically.

Please see the separate file for images. Informed consent was obtained from the patient.

## Discussion

Pilomatrixoma is an exceedingly rare cutaneous tumour that is typically firm and 0.5–3 cm in diameter. Our patient case matches some characteristics of a typical presentation; it was found on the neck, as a solitary, non-tender nodule. However, it was not firm in consistency and was quite erythematous on inspection. This reduced clinical suspicion for pilomatrixoma, which already presents a diagnostic challenge.

**Figure 1.**
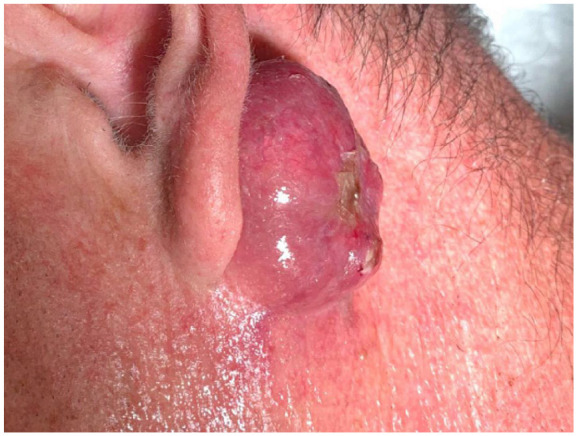
Left post-auricular fluctuant, erythematous lesion measuring 4cm with central ulceration.

**Figure 2.**
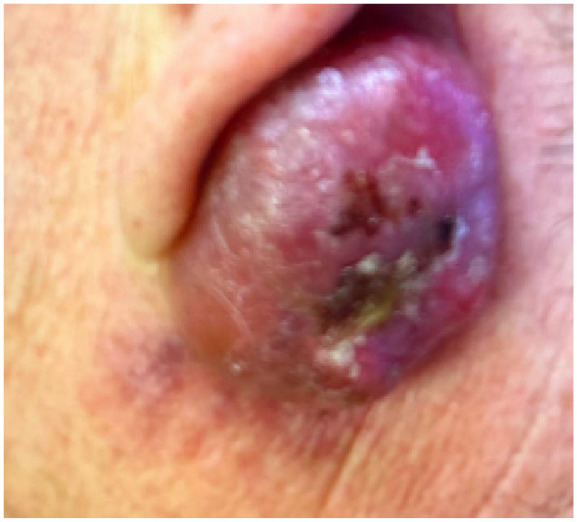
Left post-auricular erythematous lesion with central crusting and granulation tissue. Photographed 2 weeks after Figure 1.

Furthermore, the pilomatrixoma recurred and measured 4 cm (Figure 1 and 2); both of which are atypical for pilomatrixoma.^1,14,22,23^ This pilomatrixoma is among 1%–5% of all pilomatrixoma due to its recurrent nature.^1,14,22,23^ As the lesion grew to 4 cm in diameter, it is considered a giant pilomatrixoma. This further increases the possibility of misdiagnosis. Moreover, a large lesion increases the index of suspicion for malignancy. As a result, this increases the likelihood of unnecessary investigations and interventions.

Our case report presents the case of a cystic pilomatrixoma. This is a rare variant of pilomatrixoma. A recent study, by Sung et al., finds that, among the variants of pilomatrixoma, such as malignant, proliferative and bullous, the cystic variant is one of the least investigated, with only three previous reports of cystic pilomatrixoma.^28^ This, in turn, results in reduced recognition of a subtype of pilomatrixoma, where there is already a large gap between diagnosis and incidence rate. Our patient was seen for a second opinion by another plastic surgeon in the clinic after recurrence of the lesion. This surgeon described the lesion as an atypical variant, specifically referring to its soft consistency. The lack of firm consistency poses a significant concern for misdiagnoses. This has previously been addressed by Kakarala et al.^7^

The combination of these atypical characteristics may confound clinicians. As a result, this complicates the clinical investigative process and increases the chances of misdiagnoses. Furthermore, certain characteristics, such as intense erythema and giant form, increase the index of suspicion for malignancy and result in unnecessary investigations and undue stress for patients.

## Conclusion

There is a paucity in the literature on cystic pilomatrixoma. This study highlights variance in the clinical picture of pilomatrixoma, with the aim of improving clinicians’ awareness of and, in turn, clinical sensitivity to, pilomatrixoma and its diverse presentations.
